# Residents’ perceptions of radon health risks: a qualitative study

**DOI:** 10.1186/s12889-019-7449-y

**Published:** 2019-08-14

**Authors:** Selim M. Khan, Samia Chreim

**Affiliations:** 1 Interdisciplinary Population Health Doctoral Program, Faculty of Health Sciences, 25 University Private, Ottawa, ON K1N 6N5 Canada; 2 Telfer School of Management, University of Ottawa, 55 Laurier Av. East, Ottawa, ON K1N 6N5 Canada

**Keywords:** Radon, Risk perception, Testing, Mitigation, Enabler, Barrier, Health communication, Health policy

## Abstract

**Background:**

Radon is a high impact environmental pollutant and is the second leading cause of lung cancer in Canada. Building design, extended winter, and geographical location expose residents of Ottawa-Gatineau (the national capital region in Canada) to an increased risk. It is surprising that residents have an inadequate awareness of the risk - despite its gravity - and have taken minimum preventive actions. This study explores perceptions of radon health risk and examines the factors that enable and hinder the adoption of preventive measures among Ottawa-Gatineau residents.

**Methods:**

We conducted semi-structured interviews with 35 residents with varying educational and income levels to inquire about their knowledge and perception of radon, and to explore their views of enablers and obstacles to taking action to reduce radon risks. Thematic, inductive data analysis was undertaken.

**Results:**

The results indicate that: 1) Residents obtained information on radon from various sources that include the media, their education or occupation, their social network, and home renovation events. Limited references were made to the National Radon Program responsible for testing for radon and informing residents. 2) Awareness of radon risk varied, and the knowledge retained by some residents is insufficient to adequately protect their health. 3) Enablers for taking protective action included: having an understanding of the risk along with health consciousness; caring for family and children; knowing others who had contracted lung cancer and having financial resources. Obstacles consisted of: lack of awareness; cost; lack of home ownership; and potential difficulty in selling the house. 4) Residents attributed primary responsibility to public agencies for disseminating information, and incentivizing or mandating action through more stringent regulation.

**Conclusion:**

Risk perceptions are subjective, and are influenced by micro and macro level factors. Inducing protective action to reduce risk requires comprehensive interventions taking into account the dual cognitive and emotional aspects of risk perception. Future research may explore the dual aspects of risk perception and examine the contents of the risk communication message. Policy should address the responsibility of both governments and residents in tackling the issue.

**Electronic supplementary material:**

The online version of this article (10.1186/s12889-019-7449-y) contains supplementary material, which is available to authorized users.

## Background

Radon is a ubiquitous environmental pollutant that poses a significant health risk, especially in cold countries [1]. In Canada, radon kills at least 3000 people annually, making it the second leading cause of lung cancer [2]. This naturally-emitted soil gas enters buildings, typically by seeping in through foundations [1]. Building design, extended winter, and geographical location expose residents to increased concentrations of radon gas [3, 4]. Radon decay gives off tiny radioactive particles that, when inhaled, can cause mutation in DNA of lung cells eventually resulting in lung cancer [5, 6]. Women and children appear to be more strongly affected [7], and the risk is up to threefold for smokers [8].

The National Radon Program (NRP) of Health Canada is responsible for raising public awareness through disseminating radon health risk messages [9]. Despite multiple efforts, the program lags behind the desired public uptake. According to the latest national survey, 55% of Canadian households heard about radon, but only 6% of them tested for it [2]. Similarly, a recent survey conducted in the national capital region in Canada, which is known for relatively elevated levels of radon, revealed that while 32% of those surveyed expressed some concern about radon health risks, only 12% tested for radon, and 3% took action to reduce radon health risks [10]. The gap between risk awareness and actual testing rates presents a challenge for public health professionals [11, 12].

In Canada, like many other countries, residents are responsible for radon testing and remediation [1, 9]. The guideline issued by the federal government is voluntary; it assumes that residents will act rationally according to the provided information [9]. This assumption, however, has been challenged by previous psychological and preventive health research relating to health risk perception [13, 14]. Further, Spiegel and Krewski [11], in a quantitative study, assessed the health risk perception of residents in high radon areas and concluded that the Canadian residential radon exposure guideline had not been effective at prompting homeowners to take action to reduce exposure.

The term ‘risk’ denotes the probability of experiencing a dangerous event and the magnitude of the consequences of that event [15]. However, studies over the decades have provided convincing evidence that subjective perceptions of risk differ significantly from the scientific evaluation of risk [16–18]. For example, in a high seismic zone, the risk that a resident will die in an earthquake may be mathematically lower than the risk of dying in a car accident. Yet, residents are comfortable with driving but panicked of an earthquake [19]. This is because residents do not mathematically or objectively calculate the risk. Slovic argues that no risk exists independently of human thoughts and culture [20]. Attempts to reduce health risks would benefit from an understanding of people’s views. Research has identified that the success of any population-level awareness program is contingent on the views and actions of key decision makers at the household level [21]. Therefore, it is important to understand residents’ knowledge and views of radon health risk.

Most studies that assess environmental health risk perceptions in Canada adopt quantitative approaches. To our knowledge, no qualitative study has been conducted that examines residents’ knowledge and understanding of radon health risks, their views of enablers and obstacles to testing and mitigating, and their suggestions regarding how to improve the public’s knowledge and action with respect to radon. Qualitative research enhances understanding of people’s perceptions and experiences and is well suited to capture these views and their context in people’s own words [22, 23]. It is important to achieve an understanding of residents’ views. Therefore, this qualitative study focused on three research questions:

1. *How do residents learn about radon and how do they view the risks associated with it?*
2. *What are the enablers and barriers to mitigation of the risk according to residents?*
3. *What suggestions do they provide to enhance information dissemination and protective action?*

## Methods

### Sampling and data collection

This qualitative study follows a quantitative study we previously conducted with residents of the Ottawa-Gatineau area [10]. In that study, we asked respondents to provide their email if they were willing to receive future correspondence; 286 individuals provided their email. Of these individuals, we purposefully selected 32 who had shown at least some background knowledge of radon. They included: homeowners and tenants; individuals who had tested and not tested homes for radon; and individuals with various educational and occupational backgrounds. We also included in this study three interviews conveniently sampled for a pilot study on the same topic. In total, we conducted thirty-five interviews. Table 1 presents study participants’ sociodemographic characteristics.

**Table 1:** Sociodemographic characteristics of participants

Characteristics	Numbers	Percentage
Gender		
Female	8	23%
Male	27	77%
Age Groups		
18–44	7	20%
45–64	15	43%
65+	13	37%
Level of Education		
High School	3	9%
College	9	26%
Bachelor	11	31%
Graduate	12	34%
Total Household Income		
Less than $40,000	3	9%
Between $41,000-75,000	7	20%
Between $76,000-100,000	9	26%
Between $101,000-150,000	11	31%
Between $151,000 and Above	2	5%
Prefer Not to Answer	3	9%
Homeownership		
Homeowner Tenant	29 6	83% 17%

The interview guide included both closed and open-ended queries on sources of information on radon, views on radon health risk, and enablers and obstacles to taking action related to radon. We asked residents to provide suggestions on how awareness of radon health risks and actions to reduce such risk could be achieved. The first author conducted all the interviews, conferring periodically with the second author to update and discuss progress. All interviews were digitally recorded and then transcribed.

### Ethical approval

Our research complied with Canada’s Tri-Council ethics guidelines. The University of Ottawa Research Ethics Board ‘Health Sciences and Science REB’ approved the study protocol (H10–17-03). We secured informed written consent from all respondents for participation as well as for voice recording and direct quoting. We assigned a number to each participant to maintain anonymity and excluded identifying information from the quotes.

### Data analysis

We adopted an inductive approach to the analysis and focused on understanding issues from the perspective of residents. Following Braun and Clarke [24] and Miles et al. [23], after familiarization with the transcripts, initial codes that were close to the data were applied. The first author initially coded seven interviews, using descriptive codes. The process of coding was iterative: as this coding progressed, new codes were added, and some codes were modified. Following this step, the second author reviewed the coded interviews, and the two researchers developed a code list [24] that was used to recode the interviews. The first author then continued coding the other transcripts, while also convening with the second author on a regular basis to discuss emerging patterns in the data. These patterns or themes were developed in answer to the research questions. For example, initial descriptive coding indicated that some participants had learned about radon from newspapers, radio, TV and/or the internet. Each of these four had its own code, and these codes were then combined under the theme of “media sources,” which was one of the themes helping us to answer the research question on sources of information on radon. Our final themes and sub-themes are presented in Table 2.

**Table 2:** List of themes and associated sub-themes

	Themes	Sub-themes
1	Source of knowledge	Media Social network Education and occupation Home renovation shows Home inspectors The current study National Radon Program
2	Knowledge-risk awareness	Advanced Basic Deficient Faulty
3	Enablers	Understanding health risk (cognitive awareness) Concern for children and oneself (emotional awareness) Social influence (knowledge of significant others) Personalizing the risk Financial capability
	Barriers	Lack of awareness of health risk Cumbersome procedures Cost of remediation Tenancy Reduced property value (assumed) Voluntary nature of the guidelines
4	Residents’ suggestions for improving knowledge dissemination and protective action	Role of public agencies Individual responsibility Shared responsibility Role of citizenry Incentivizing Mandating and legislating

## Results

In this section, we report the results of our analysis of the sources of information and level of knowledge of participants. We then present participants’ views of enablers and obstacles to testing and mitigation. This is followed by a summary of participants’ suggestions regarding ways to improve disseminating information and taking action to reduce radon harm.

### Source of knowledge

We sought to understand how participants gathered knowledge on radon – their source of information and how they came upon it. We found that residents learned about radon through information gained from various sources, the most common of which were various types of media such as newspapers, television, radio and the internet:

“There was a report in the Ottawa Residents, a local newspaper in the Kanata area” (SP19).

“I read something about it years ago, in the Maclean’s magazine” (SP18).

“I watch television programs. When I was listening to the news, I heard them talk about radon” (SP20).

“I read some articles online” (SP22).

Other less common sources included one’s social network of friends and clubs:

“We had friends who live a few kilometers from here, who told us about radon. This increased our awareness” (SP1).

“A lot of my friends used to work in Health Canada; I heard from them” (SP35).

“We were told in a geological club to be careful of some of the rocks … that one of the byproducts of that was radon gas” (SP24).

A few participants mentioned their own occupational education and practice as sources of information:

“I was trained in hazardous materials operations; so, I came to know about radon gas” (SP32).

A few participants learned about radon in the course of considering home renovation, construction or real estate transactions:

“I first heard about radon about 15 or 20 years ago when I was planning to set up a marble countertop, and the marble countertop scheme was up” (SP18).

“Several years ago, I attended a home and cottage show here in Ottawa, and I had to stop in a stall that I believe was by the National Research Council. There I learned about radon. That was the first source of information about radon for me. I then looked through the internet. I read widely” (SP16).

A source of information that one would expect to be highly salient is *home inspectors* because these professionals are typically hired by new homeowners or residents to advise them on construction and other dwelling-related problems. However, only one participant mentioned this source of information:

“When we moved here, the home inspector mentioned that radon might be an issue in this area (Kanata)” (SP34).

Perhaps most alarming was the fact that several participants indicated that they learned about radon from the current study:

“I had not heard of radon till I was contacted to participate in your survey” (SP10).

“The first thing I heard about radon was your ad; from the links that you sent to me” (SP28); (please see recruitment ad in Additional file 1).

“I heard about it from your study” (SP25).

In sum, residents obtained information on radon from various sources, but this does not tell us whether the information they obtained and retained is accurate. We consider this issue next.

### Level of knowledge and awareness of risk

Participants had different levels of knowledge about radon gas, its source and its potential effects; thus, awareness of risk varied across respondents. Some had very advanced knowledge and awareness of the risk, which was associated with their education or occupation. For example, the three participants below had backgrounds or occupations in biology, construction consultancy and engineering respectively:

“I have a biology background … I know how the situation can arise in a building, and what conditions generate the gas” (SP17).

“I have some knowledge about uranium degradation. In a climate such as this, the effects are worse in the winter because the house tends to be closed up … I think radon is quite serious as it is odorless, you don’t know about it unless you test for it, so it kills people silently, and that’s why it’s serious” (SP29).

“70 Bq (Becquerel) is equal to one X-ray. We had 280 Bq - that is equivalent of basically 4 X-rays in a year” (SP24).

Others’ advanced knowledge was based on their motivation to learn more about radon’s health effects in order to reduce the health risk. For example, a study participant stated:

“The best time to test for radon is in the winter when the doors and windows are kept closed. In summer, we usually keep our doors and windows open and so the gas does not build up. There is variation in radon release. It varies even on a diurnal basis. There are other factors that impact radon release, so it is good to test often, and even better to have a real-time detector if you have the capacity to buy one” (SP11).

Individuals with advanced knowledge also showed understanding of the synergistic risk associated with smoking:

“Radon is present everywhere. It is the level of radon that is harmful. You should know what the level is. If it is within the guideline level, then, we can say that there is not much harm to your health … If you have multiple potential risk factors for the same disease, such as smoking and radon in this case, then the combination of the effects is probably more than the effect from a single factor” (SP16).

Other participants had some basic facts, such as:

“I know it is a colorless, odorless gas, it seeps into the house through the basement, it is a product of deterioration of uranium. It is naturally occurring and it happens everywhere” (SP13).

However, other residents held views that were scientifically unfounded. Some of these views were that new houses are safe, that radon is not a problem in Ottawa, and that there is insufficient evidence on the harms of the gas:

“New houses have no radon: I suspect that my house does not have radon. Because of the age of the house – it is not ten years yet” (SP27).

“Houses built on clay, no chance of having radon” (SP26).

“It’s not a problem in Ottawa but in Winnipeg and Western Canada” (SP35).

“I don’t think there are sufficient studies to categorically say that this is serious enough” (SP20).

In sum, some of our participants did not specifically seek information, and were rather passively exposed to it through the media or by hearing from colleagues, while others took a more active stance, seeking advanced information. Awareness of risk varied, and it is important to note that the knowledge retained by some residents is faulty and potentially harmful to their health.

### Enablers and barriers

We asked participants about the factors that would facilitate or hinder radon testing and preventive actions. Enablers to taking protective actions included: having an understanding of the risk, being health conscious, being responsible for providing care for family and children, having knowledge of others who had contracted lung cancer, and having financial capability. The obstacles consisted of lack of awareness of health risk, cumbersome procedures, cost, lack of home ownership, concerns about reduced property values, and the voluntary nature of the guidelines.

#### Enablers

Most of the participants who indicated that they engaged in actions aimed at testing for radon levels and/or reducing the risk of radon showed a clear understanding of the risk (cognitive awareness) and had also concerns (emotional or affective awareness) regarding their family members’ health – especially worries about children’s health. These individuals were most motivated to take action.

“My daughter has been sleeping in the basement for thirteen years; I wanted to know whether she has been exposed to radon for this long time” (SP29).

“My son used to sleep in the basement from time to time” (SP32).

“My daughter does sleep there quite often; so, I am concerned about her sleeping in the basement and a buildup of radon” (SP23).

Attention to and concern regarding one’s own health was also mentioned as an enabler:

“It's the concern about having lung cancer from radon. I am health conscious; if I know something is a risk factor, I would go for fixing that” (SP7).

“The desire to avoid any unnecessary health effects. There are health hazards while we drive on the highway and that’s why you make sure that your vehicle is in good shape and you also put on seat belts. The same kind of thing applies here. If you are aware of the health hazards, you take the appropriate measures*”* (SP16).

“It was the concern about my health that encouraged me to test and fix the house for radon … I could not afford any more insult (exposure to radon) to my lungs” (SP24).

Knowledge of others who had contracted lung cancer seemed to influence some participants to take action. Specifically, seeing individuals who could be similar to oneself and who had contracted lung cancer, heightened risk perception (i.e. personalizing the risk through a vicarious experience):

“There is a man of 60 in my neighborhood who got lung cancer” (SP30).

“We have friends who live a few kilometers from here, who told me that he was concerned about his home and he tested … I was surprised because my son used to live in the basement and so my husband became concerned when he got the information” (SP1).

“I was watching a medical program on TV that was doing a feature about women and cancer, specifically lung cancer. The women in the program who got lung cancer did not smoke. They had a healthy lifestyle, they were fit, they exercised, but they still got lung cancer. So, the doctor was trying to find out –how was that possible?... The cancer researcher, I don’t recall his name, said that there is a high possibility that it was radon gas … So, they recommended to stay healthy, to take action, and directed to have the house tested to see if there was radon gas” (SP7).

Another enabler was financial capability. Given that residents typically bear the cost of testing homes and of mitigating for the potential harm, being financially capable appeared as an enabling factor:

“Certainly, it would be expensive for people who are not as fortunate as we are” (SP1).

“I don’t think it is that much expensive, this is not a barrier for me” (SP2).

#### Barriers/challenges

Although none of the participants indicated that lack of awareness was an obstacle to taking action for themselves, most referred to this factor as a potential barrier for others to take action:

“First is knowledge, if people don't know anything at all – it's the first challenge” (SP11).

“I think most people are not aware of what the indoor air quality is in a house” (SP21).

The cost of mitigation also came up as a barrier to taking action, especially with respect to reducing radon levels in a home, and less so, with respect to purchasing a monitoring device:

“There is always the finance involved because one has to bring in a specialist and it may cost up to $4,000” (SP7).

“I just cannot afford it” (SP20).

One participant spoke about the various procedures associated with testing as a potential barrier, indicating that the process may be cumbersome for some residents who might not have enough time or patience:

“Other things may be where to get the kit, not knowing how much it may cost and how to do the test. There are the procedures to place the test kit at a certain place and wait for over 90 days and then send it to the lab by mail and wait for the result. These all take time … It also requires the willingness to go ahead and follow through with all the steps till getting the result. Some people may not have that time and patience” (SP16).

Lack of home ownership – tenancy – was mentioned as a potential barrier:

“If I am a renter and my landlord is not aware of this problem, he may not do the test” (SP5).

“ … for some, not owning the home - like tenancy, people who rent their houses” (SP15).

“If you are renting your house and do not own it, then it may be a challenge” (SP25).

Another challenge mentioned by homeowners was the difficulty in selling the house should radon levels be tested and found to be elevated:

“People may think that if radon is detected, it may be a problem in selling their houses. There is a stigma associated with this - that the house is contaminated. In areas where these types of contaminants are found, assessed property values tend to decrease” (SP24).

“ … because otherwise, they lose the value once they are going to sell the house” (SP32).

Reference was also made to the voluntary nature of the guideline as a potential barrier for mitigation:

“If the building codes are voluntary and it costs even $100, nobody is going to do it as that means paying $100 from the builder’s pocket. But if it is mandatory, then only it will be done” (SP32).

“I think almost everything that is voluntary is not going to motivate people to take action. If it’s mandatory, then people will follow the guideline, even if they are not well aware of the issue” (SP33).

In sum, various enablers and barriers influence residents’ action-taking with respect to radon.

### Residents’ suggestions for improving knowledge dissemination and protective action

Given the limited information that some participants had - and the fact that some of the knowledge they retained was not scientifically-based and could potentially be harmful - we sought to understand participants’ views of what should be done to raise residents’ awareness of the health risks and encourage them to take action. We believed it was particularly important to obtain residents’ views, given that there are national guidelines in place, yet these do not appear to be highly effective in educating the public or incentivizing it to take action [2, 10]. This indicates that the program is poorly matched with most residents’ needs for information and incentive. It was, therefore, particularly important to learn from residents what might be helpful for them.

We, therefore, asked participants to provide suggestions and recommendations on courses of action to help raise awareness and take action, and found out that the greatest majority of participants emphasized the role of public agencies. References were made to concerted action including “the government at all three levels – municipal, provincial and federal” (SP15) in disseminating information about the presence and health risks of radon, and to seeking participation from “all the stakeholders who may be involved in this issue” (SP2), including the government, the private sector (e.g. the homebuilders) and associations (e.g. the Cancer Society). The view was that this should be done through various media. Participants stated that a credible government body such as Health Canada should engage in effective publicity campaigns using print health education materials (such as pamphlets providing information) and audio-visual media such as TV. Some suggested specifically broadcasting through local news channels, and others suggested adopting a combination of approaches:

“In television, there should be an image of some of the effects of radon, and it will be easier for people to understand” (SP25).

“One option could be sending a flyer to the mailbox as the hydro company does for energy efficiency, could be a newspaper article, could be online, could be television shows … or it could be a mix of all these things as different people have different choices and means to get the information” (SP29).

The very few residents who focused on the role of the individual either referred to individual harmful behaviors or believed that the individual should take action after being informed by the government:

“Well, it could be the individual’s problem, it depends on a lot of things like people’s living habits. Some people smoke … that could contribute to that. So, this is an individual rather than an overall societal problem. In that particular case, the responsibility should go to the individuals, not to the government” (SP35).

“I think the responsibility goes primarily to the individuals. The government has the role of providing information, but it is the individuals who have to take steps to test and where necessary, to mitigate the issue” (SP16). These views were few in number.

Others indicated that “it is a shared responsibility” (SP34), stating that:

“It's a societal problem, not individual because it runs from coast to coast” (SP7).

“I think it is an overall social problem, as it affects the health of everybody” (SP30).

According to them, the governments should be responsible for informing people about the risk but the individual property owners should take the initiative for testing, and where bearing the mitigation cost exceeds the financial capacity, one could seek support from the government:

“I think both have the responsibilities. Government can support, but homeowners should take the initiatives to do the test first” (SP11).

“The initiative should be taken by the homeowners; the government should be in support (SP12).

There was also the suggestion that the use of celebrities may help drive the message more strongly:

“You can get help from David Suzuki; he would be the best person to communicate this message” (SP15).

Getting help from health clinics to disseminate the message was also offered as a suggestion:

*“*The clinics can do this job by putting up signs that draw people’s attention” (SP1).

Two participants emphasized the role of the citizenry and the communities in raising awareness through processes similar to social movements:

“It is not your issue or my issue, it is everybody’s issue, and we have to get together to stop this from happening” (SP20).

“People from the radon-prone areas should come forward” (SP13).

Suggestions regarding actions aimed at testing and/or mitigating were framed in terms of incentivizing and/or legislating procedures. For example, some participants stated that providing free test kits, tax rebates and subsidies would encourage some residents to take action given that “most people are not going to pay for the test. If someone has a minimum wage and you think he is going to test and fix his house by himself- it’s not going to happen” (SP27).

While these suggestions address incentivizing procedures, other suggestions focused on legislating and setting mandatory requirements. A few suggestions centered around making testing mandatory, though it was also recognized that this might be difficult to achieve. Other areas where government legislation was seen as more effective was in terms of setting building codes:

“The government needs to put in place building codes -every house built in such a way that a minimum requirement has to be met to prevent radon gas” (SP24).

“If there are CO (Carbon Monoxide) and smoke detectors in houses, why not a real-time radon detector?” (SP30).

Participants also suggested legislating real estate transactions and including a mandatory provision for property sellers to declare the radon status during all real estate transactions:

“Testing and disclosure – both should be made mandatory” (SP34).

“I would be very upset if I find after the fact that there is radon in the house. I think it’s reasonable to have a clause in the sale agreement” (SP32).

Thus, the recommendations focused mainly on the role of public agencies in disseminating information, and in incentivizing or mandating action through more stringent regulation.

## Discussion

The objectives of this qualitative study were to gain an understanding of residents’ knowledge and perceptions of the risk associated with radon, to identify the enablers and obstacles to taking action, and to obtain their suggestions regarding how knowledge and action associated with radon can be improved. The results showed that residents acquire knowledge passively or actively from diverse media sources, their social network, their education and/or occupation, home shows and home inspectors. Most surprising was the fact that several residents came to know about radon for the first time from the current study. This supports the findings of our preceding study [10] and the conclusions of previous studies that the Canadian National Radon Program has not been highly effective in informing residents and in prompting action to reduce radon exposure [11].

While most of the residents had basic knowledge about radon, a few demonstrated in-depth knowledge. However, an important result from this study is that some residents have misconceptions about radon, showing what has been termed unrealistic optimism, which involves individuals holding an unreasonably low estimate of their own susceptibility to the risk [16]. Studies in the US [17] and Ireland [25] have demonstrated that it is not uncommon for individuals living in high radon areas to underestimate the potential harm or to believe that radon is a threat to others but not to themselves.

Among the accounts from participants were references to a 60-year old neighbor, and to women in a TV show who were otherwise healthy and non-smokers, and who had contracted lung cancer. The availability of examples of harm may allow one to form a judgment that radon poses a health risk [13]. Still, caution should be taken in assuming that merely being exposed to the risk information can lead to the formulation of appropriate conclusions or to taking appropriate action. Researchers [16, 26] have shown and argued repeatedly that information processing and behavior are highly complex and are influenced by multiple factors at various levels. For example, Weinstein et al. [26] stated that “an intervention capable of having a major impact on perceptions of risk may have to confront directly the mini-theories people construct to justify their optimism” (p.32), thus pointing to *micro-level* factors. The authors note that such interventions would be challenging to implement on a large scale given the various mini-theories that people construct [26]. Specifically, in our study, the findings show that associating radon health risk with both personal (e.g. diagnosis of lung cancer) and family members’ (especially children’s) health appeared as an impetus for residents to test, mitigate and continuously monitor radon level. These are examples of events and occurrences that positively helped to shape individuals’ mini-theories.

The presence of such mini-theories suggests that the more varied the media, the more diverse the types of messages (e.g. cognitive and emotional appeals), and the more agencies delivering the message (e.g. different levels of government, associations, health clinics), the higher the likelihood that knowledge and action regarding radon will increase. Studies in the UK have shown similar results where multiple radon health risk communication campaigns effectively raised awareness [27], and health communication messages from authentic sources had positive effects especially in radon-prone areas [28].

As we have pointed out, risk perception is a subjective phenomenon. It is influenced not only by micro-level factors such as individual mini-theories and information processing but also by the macro-level context [29]. At a *macro-level*, we encounter the social context of radon threat, where views of risk and of who is responsible for reducing the risk are influenced by the community or social framing of the issue [29]. For radon, there is no collective community action built through consensus, and therefore, individuals show various views. Whereas some established social norms, consensus and groups (as on gun control in the US) can motivate people to act, the absence of such a community movement influences inaction even in high radon areas [30]. In this vein, a few participants in our study called for residents to get together and to engage in collective action on the issue.

Another macro-level factor of relevance in interpreting the results of our study is that the health care system in Canada is publicly-funded and highly regulated. Federal, provincial and municipal government bodies have extensive regulations associated with various health risks. This helps account for residents’ views regarding the role of government in managing radon risks. The majority of participants viewed government as the primary actor with responsibility for informing the public and for incentivizing and mandating action to reduce radon risks. This points to the critical role that health policy can play in reducing risks.

The suggestions we received from the participants pointed to the need to involve various public and non-governmental agencies, various types of messages, and various incentivizing and regulatory steps. This is particularly important because research has shown that simply providing information is not sufficient [31]. For example, evidence has shown that when a health message is personally significant, people try to moderate the seriousness of the health risk, raise questions about the accuracy of the risk information, and process it in a subjective manner [30, 32, 33].

The primary audience of radon messages is the at-risk population who live in an area with high levels of radon. This segment could be challenging to persuade because they might process the information defensively when it is too intimidating [33]. Defensive techniques include, for example, avoidance of message [34] and denial of susceptibility [32]. However, there is also evidence showing that individuals’ motivation for action is driven more by the emotional aspects of perception than by cognition [10, 31, 35]. These various considerations reinforce the importance of using different approaches and messages.

Our study did not gather systematic information from participants about the content of messages on radon that they would find to be effective, as this was outside the scope of the study. However, some of the participants’ accounts indicated that the content is important. In fact, some research has indicated that individuals are likely to feel more threatened by a description that assigns an active rather than a passive agency to radon (e.g. ‘radon gas invades your home’ as opposed to ‘radon gas seeps into homes’) [36]. Therefore, studying residents’ views of message content would help improve communication campaigns on radon.

### Limitations

A limitation is our inability to generalize from the current study to the population at large; however, this is a typical feature of qualitative studies [22]. In fact, exploratory qualitative studies are not intended to provide generalizable data, but to enhance understandings that can be further tested in broader-based quantitative studies. Our study was also limited in the number of participants and locations. Future research would benefit from interviewing more participants and in other cities to generate additional insights. We believe that studies in cities where there have been condensed and concerted efforts to communicate with residents and incentivize action would provide an interesting extension of or counterpoint to this study.

## Conclusion

This study sheds light on the vital topic of residents’ knowledge and views of radon and its risks. To date, studies on views of radon risk in Canada have tended to adopt quantitative approaches. The value of the qualitative approach is that it provides a deeper look into participants’ experiences, thus providing information to complement quantitative results. Our study showed that many people do not get the crucial information on radon and some retain misconceptions about the health risks of radon even after being exposed to information. Many residents report barriers in testing and engaging in protective action, even after receiving the pertinent information.

Our study underscores the importance of seriously considering how radon risk is understood and dealt with by residents. We identified that mere cognitive risk awareness is not enough; additional emotional awareness motivates residents to take action regarding testing and mitigating their houses for radon. Therefore, radon health communication will be more effective when programs address both these aspects of risk perception along with plausible regulations and incentives, where necessary. Future research can explore the dual aspects of risk perception and thus help to develop tailored risk communication messages capable of motivating residents to mitigate their homes for radon health risk.

## Additional file

Additional file 1: Recruitment ad. (PDF 104 kb)

## Data Availability

Available from the corresponding author on reasonable request.

## Abbreviations

NRP: National Radon Program
SP: Study Participants
