# Supplementary material for: Residents’ perceptions of radon health risks: a qualitative study
Source: BMC Public Health. 2019 Aug 14;19:1114. doi: 10.1186/s12889-019-7449-y (PMC6694642; doi:10.1186/s12889-019-7449-y)
Supplement: Supplementary file 1 — Recruitment ad. (PDF 104 kb) [file 12889_2019_7449_MOESM1_ESM.pdf]

Looking for research participants!

Why are we doing this study?

- Canadians spend over 90% of their time indoors.
- Indoor air quality is crucial for our health, more so for our children
- The study aims to explore your perception about indoor radon gas that will help support policy

Who can participate in this study?

- Homeowners and tenants from Ottawa-Gatineau and neighborhoods
- Living in a house for at least one year that has a used basement or living space on the ground floor

How, where and when?

- Face to face interview
- At your preferred place and time

What do you do?

Participate in an interview that takes about 45-60 minutes

How to join the study?

Call to: xxx or email: skhan196@uottawa.ca

What will I get?

•Acquire knowledge about indoor air quality, risks and solutions
•Satisfaction from contribution to knowledge generation
•A free test kit is available to voluntarily test your house for radon
